# Impact of the COVID-19 pandemic in the treatment of patients with acromegaly in a tertiary center: a wake-up call on the importance of telemedicine

**DOI:** 10.20945/2359-3997000000491

**Published:** 2022-06-27

**Authors:** Rafaela de Jesus Nunes, Lais Farias Masullo, Matheus Zaian Rodrigues de Fonseca Lira, Cíntia Maria Gomes Leite, Thierry Mendes Gomes, Eveline Gadelha Pereira Fontenele, Ana Rosa Pinto Quidute, Manoel Ricardo Alves Martins

**Affiliations:** 1 Universidade Federal do Ceará Programa de Pós-graduação em Patologia Fortaleza CE Brazil Programa de Pós-graduação em Patologia, Universidade Federal do Ceará, Fortaleza, CE, Brasil; 2 Universidade Federal do Ceará Programa de Pós-graduação em Desenvolvimento e Inovação Tecnológica de Medicamentos Fortaleza CE Brazil Programa de Pós-graduação em Desenvolvimento e Inovação Tecnológica de Medicamentos, Universidade Federal do Ceará, Fortaleza, CE, Brasil; 3 Centro Universitário Christus (Unichristus) Fortaleza CE Brazil Centro Universitário Christus (Unichristus), Fortaleza, CE, Brasil; 4 Universidade Federal do Ceará Fortaleza CE Brazil Graduação em Medicina, Universidade Federal do Ceará, Fortaleza, CE, Brasil; 5 Hospital Universitário Walter Cantídio Fortaleza CE Brazil Serviço de Endocrinologia e Diabetes, Hospital Universitário Walter Cantídio, Fortaleza, CE, Brasil; 6 Universidade Federal do Ceará Departamento de Fisiologia e Farmacologia Fortaleza CE Brazil Departamento de Fisiologia e Farmacologia, Universidade Federal do Ceará, Fortaleza, CE, Brasil; 7 Universidade Federal do Ceará Núcleo de Pesquisa e Desenvolvimento de Medicamentos Fortaleza CE Brazil Núcleo de Pesquisa e Desenvolvimento de Medicamentos, Universidade Federal do Ceará, Fortaleza, CE, Brasil; 8 Universidade Federal do Ceará Departamento de Medicina Clínica Fortaleza CE Brazil Departamento de Medicina Clínica, Universidade Federal do Ceará, Fortaleza, CE, Brasil

**Keywords:** Acromegaly, COVID-19, telemedicine, coronavirus

## Abstract

**Objective:**

The COVID-19 pandemic has profoundly disrupted health care worldwide. We aimed to evaluate the impact of the first COVID-19 wave on the treatment of our patients with acromegaly.

**Subjects and methods:**

A standard questionnaire was systematically applied to all patients and included questions on general health status, whether all laboratory workup had been done, common signs and symptoms of acromegaly, treatment adherence, and previous COVID-19 symptoms and diagnosis.

**Results:**

We attempted to contact 136 patients with acromegaly at regular follow-up at our institution and contacted 101 of them successfully. In all, 37% of the patients reported symptoms of acromegaly, which was more common among women. A total of 27 patients were lost to follow-up (including 19 who interrupted treatment during the pandemic) mainly for fear of becoming infected by the SARS-CoV-2. Of these, 24 resumed follow-up after our contact.

**Conclusions:**

The current COVID-19 pandemic has strongly impacted the follow-up of patients with acromegaly. Telemedicine can be an important tool to maintain regular treatment in the current or future pandemics.

## INTRODUCTION

The current COVID-19 pandemic has profoundly disrupted health care worldwide. However, the impact of the pandemic is not restricted to the consequences of the virus *per se* , as the pandemic may also lead to death from overlooked diseases and disrupt the follow-up of patients receiving care ( 1 ).

Acromegaly is a debilitating disease secondary to a chronic excess of growth hormone (GH), usually caused by a pituitary adenoma. The disease is associated with increased morbidity and mortality. Control of excessive hormone secretion in acromegaly has been widely shown to decrease patients’ mortality ( 2 ). Hence, delayed or interrupted treatment may have adverse health effects ( 3 ).

The COVID-19 pandemic may have substantially impacted patients with acromegaly by increasing obstacles to regular follow-up visits and adherence to medical treatment. Due to high costs, both clinical treatments of choice for these patients (the somatostatin analogues octreotide and lanreotide) and the second-line clinical treatment (cabergoline) are offered by the Brazilian national public health system ( *Sistema Único de Saúde* – SUS) ( 4 ). After evaluation by the physician, patients for whom these clinical treatments are recommended must appear in person to pick up their medications at a specialized pharmacy, bringing along their prescription and documentation. Dopaminergic agonists are available as pills, but octreotide and lanreotide are only available for parenteral injection (intramuscular and subcutaneous, respectively). Notably, lanreotide can be administered by the patient or a relative (although self-administration is not currently typical in Brazil), while octreotide requires a health care provider with expertise in administering this drug ( 4 , 5 ).

Our center’s elective outpatient care was reorganized during the pandemic following local government recommendations. Although the medications continued to be regularly dispensed, many factors contributed to decreasing patients’ adherence to treatment, including fear of leaving home and becoming infected and decreased availability of public transportation.

As reported by health authorities, the peak of the first COVID-19 wave in our state occurred between late April and early May 2020. Based on that, the aim of this study was to evaluate the impact of the first COVID-19 wave on the care of patients with acromegaly following up at our center.

## SUBJECTS AND METHODS

A standard questionnaire was prepared and systematically applied to 101 patients with acromegaly undergoing treatment at our tertiary reference center from July to September 2020 ( Appendix A ). Telemedicine consultation was delivered following recommendations by the Federal Council of Medicine. The patients were identified from the hospital’s system registration and were contacted via mobile phone. Our patients had previously shown great response in answering our calls and responding to questions by phone. An examiner verbally asked open-ended questions to the patients about their general health status, whether all laboratory workup had been done, common signs and symptoms of acromegaly, adherence to treatment, and previous diagnosis or symptoms of COVID-19.

COVID-19 indicators were obtained from the integraSUS system ( 6 ), a tool that integrates epidemiological, hospital, outpatient, administrative, financial, and planning monitoring and management systems of the Ceará State Health Department.

Data about the injection of somatostatin analogues were retrieved from the hospital’s integrated system. This system records medications released by the pharmacy department and administered by nursing staff, thus monitoring the actual application of the medication to the patient.

The study was conducted in accordance with the Declaration of Helsinki 2013 Brazilian version and was approved by the local Research Ethics Committee - CAAE (Certificado de Apresentação para Apreciação Ética) registration number: 34518920.0.0000.5045.

## RESULTS

We attempted to contact all 136 patients with acromegaly undergoing treatment at our center from July to September 2020. In all, 101 patients (62 women) were successfully contacted. The average age of the contacted patients was 56.0 ± 13.5 years.

Most patients reported feeling well (n = 64; 63.4%) and were asymptomatic. The most frequently reported signs or symptoms were body aches and headaches, more frequently reported by women ( Table 1 ).

**Table 1:** Signs and symptoms reported by patients during the study

Signs and symptoms	Women (N, %)	Men (N, %)
Body aches	9 (14.5%)	4 (10.2%)
Headaches	8 (12.9%)	2 (5.1%)
Sadness (anxiety)	6 (9.7%)	1 (2.6%)
Tiredness	4 (6.4%)	0
Abdominal pain	0	1 (2.6%)
Total number of patients	62	39

Of the 101 patients, we identified 27 (27%) who were not following up regularly at our center. Remarkably, 19 patients had interrupted follow-up during the pandemic. The main reason for follow-up interruption was fear of becoming infected with the SARS-CoV-2 (n = 17 of 19, 89.5%). All 19 patients had been prescribed clinical treatment (monotherapy with somatostatin analogues, n = 11; combination therapy with somatostatin analogues and cabergoline, n = 5; monotherapy with cabergoline, n = 2; and combination of somatostatin analogue and GH receptor antagonist, n = 1). All patients agreed to resume medical treatment. We also detected another 8 patients who had not followed up for more than 1 year due to personal reasons. Of these, 5 resumed their medical appointments after the call.

Two patients reported a positive RT-PCR test for SARS-CoV-2 coupled with symptoms of headache and body ache, while one patient reported fever, cough, and nausea. None of the patients contacted by us had progressed to a severe stage of the disease requiring hospitalization.

## DISCUSSION

To the best of our knowledge, our study is the first to evaluate the impact of the first COVID-19 wave in the follow-up of patients with acromegaly in Brazil. We found that 19% of the patients interrupted treatment during the pandemic, mainly due to fear of leaving home and becoming infected with the SARS-CoV-2.

In Ceará, the COVID-19 pandemic began in early March 2020, with the first patient diagnosed on March 15 ( 7 ). The peak of the first wave probably occurred between late April and early May 2020, and a lockdown was implemented in Fortaleza from May to June 2020 ( 8 - 10 ).

At our hospital, many health professionals were relocated to the COVID-19 unit. However, the endocrinology outpatient clinic remained open at decreased capacity. Although doctors continued to prescribe drugs, many patients faced challenges in making it to the hospital. Additionally, many patients lived outside of Fortaleza and could not use intermunicipal bus transportation due to service cancelations.

An ideal system to help patients maintain their health care needs, including their ability to undergo medical evaluation and treatment, should include telemedicine, reduced bureaucracy, digital prescriptions, home delivery of medications, and availability of health care providers to administer medications at the patients’ homes when necessary ( 11 ).

**Figure 1:**
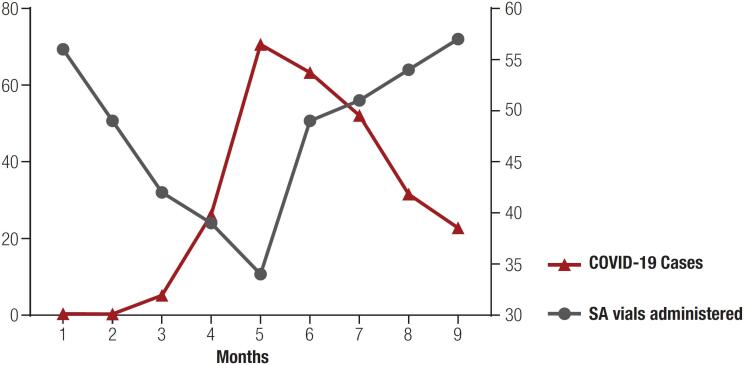
Monthly amount of somatostatin analogues vials administered in our hospital and monthly number of COVID-19 cases diagnosed in Ceará during the period of January to September 2020.

The COVID-19 pandemic has been affecting several aspects of the care of patients with acromegaly, particularly their assessment and diagnosis ( 12 ) ( Figure 1 ). In this scenario, endocrinologists have reported using telephone consultations and emails as alternative methods for communicating with their patients during the pandemic ( 13 ). In this context, telemedicine should be an appropriate means to follow-up patients with acromegaly ( 14 , 15 ). Telemedicine can complement traditional outpatient and hospital practices, creating an opportunity to implement remote monitoring and patient-centered care. This tool is vital for maintaining essential health services during the pandemic but can also become a sustainable resource for educational initiatives. Innovations in telemedicine should be designed to increase the quality of care, with safety, effectiveness, and efficiency comparable to face-to-face visits ( 16 ).

Telemedicine has been increasingly used in Brazil but has not been explored to its full potential yet ( 17 ). Barriers to the broader use of telemedicine include technological and cultural limitations. Some patients are unable to use telemedicine due to a lack of Internet connection or hardware, but telemedicine can be tailored in these situations ( 18 ). Our use of mobile phones – not only for calls but also for texting – has been highly efficient in helping our patients ( 19 ).

Bureaucracy hinders proper health care and delays treatment in some patients. In our view, bureaucracy can be reduced without decreasing the surveillance for proper use of medications.

The administration of parenteral medications is another issue that must be addressed. Efforts by public and private agents, including the availability of health care professionals to deliver and administer medications at the patients’ homes and education of the patients toward self-administration of medications, when required, are highly needed and should be encouraged.

We were unable to contact a large number of our patients. Possible reasons for this limitation include wrong contact phone numbers and screening of calls from unknown numbers. We cannot also rule out that some patients may have died due to acromegaly, COVID-19, or other diseases during this period.

The COVID-19 pandemic has strongly impacted the treatment of our patients with acromegaly. Since the pandemic is still ongoing and new pandemics most certainly will happen in the future, we want to emphasize the need for promoting actions to maintain proper patient care through these difficult times, including delivery of medications, reduced bureaucracy, and availability of telemedicine.

## Appendix A

**Table:**

	UNIVERSIDADE FEDERAL DO CEARÁ (FEDERAL UNIVERSITY OF CEARA)
	**IMPACT OF COVID-19 PANDEMIC IN THE TREATMENT OF PATIENTS WITH ACROMEGALY IN A TERTIARY CENTER: A WAKE-UP CALL FOR TELEMEDICINE.**

**Data** (date): ___/_____/_____

**Table:**

DADOS GERAIS (GENERAL DATA)	
**Nome** (Name):	
**Prontuário** (Medical record):	
**Idade** (age):	**Sexo** (sex):

**Table:**

PERGUNTAS (QUESTIONS)	
**Como você está se sentindo?**
How are you feeling?
**Quando você realizou exames laboratoriais?**
When did you perform laboratory tests?
**Quando você realizou exames de imagem (RMN)?**
When did you perform image exams (RMN)?
**Quais medicamentos está usando?**
What medications do you current use?
**Se análogos de somatostatina (Octreotida e Lanreotida), qual a data da última dose?**
If somatostatin analogues (Octreotide and Lanreotide), when was the last dose applied?
**Você está usando os medicamentos regularmente? Se não, qual o principal motivo?**
Are you using your medicines regularly? If not, what is the main reason?
**O registro para recebimento do medicamento está ativo?**
Is the registration for receiving the drug active?
**Você precisa renovar alguma receita?**
Do you need to renew any prescription?
**Qual a data da sua próxima consulta?**
What is the date of your next medical appointment?
**Você teve diagnóstico de COVID-19, através de RT-PCR? Se sim, quais sintomas relatados?**
Have you been diagnosed with COVID-19 through RT-PCR? If yes, what symptoms did you report?
